# Bayesian network imputation methods applied to multi-omics data identify putative causal relationships in a type 2 diabetes dataset containing incomplete data: An IMI DIRECT Study

**DOI:** 10.1371/journal.pgen.1011776

**Published:** 2025-07-15

**Authors:** Richard Howey, Jonathan Adam, Jerzy Adamski, Natalie N. Atabaki, Søren Brunak, Piotr Jaroslaw Chmura, Federico De Masi, Emmanouil T. Dermitzakis, Juan J. Fernandez-Tajes, Ian M. Forgie, Paul W. Franks, Giuseppe N. Giordano, Mark Haid, Torben Hansen, Tue H. Hansen, Peter P. Harms, Andrew T. Hattersley, Mun-gwan Hong, Ulrik Plesner Jacobsen, Angus G. Jones, Robert W. Koivula, Tarja Kokkola, Anubha Mahajan, Andrea Mari, Mark I. McCarthy, Timothy J. McDonald, Petra B. Musholt, Imre Pavo, Ewan R. Pearson, Oluf Pedersen, Hartmut Ruetten, Femke Rutters, Jochen M. Schwenk, Sapna Sharma, Leen M. ’t Hart, Henrik Vestergaard, Mark Walker, The IMI DIRECT Consortium, Ana Viñuela, Heather J. Cordell

**Affiliations:** 1 Research Software Engineering, Newcastle University, Newcastle upon Tyne, United Kingdom; 2 Population Health Sciences Institute, Faculty of Medical Sciences, Newcastle University, Newcastle upon Tyne, United Kingdom; 3 Research Unit of Molecular Epidemiology, Institute of Epidemiology, German Research Center for Environmental Health, Helmholtz Zentrum München, München, Germany; 4 Department of Biochemistry, Yong Loo Lin School of Medicine, National University of Singapore, Singapore, Singapore; 5 Institute of Experimental Genetics, German Research Center for Environmental Health, Helmholtz Zentrum München, München, Germany; 6 Institute of Biochemistry, Faculty of Medicine, University of Ljubljana, Ljubljana, Slovenia; 7 Novo Nordisk Foundation Center for Basic Metabolic Research, Faculty of Health and Medical Sciences, University of Copenhagen, Copenhagen, Denmark; 8 Oxford Centre for Diabetes, Endocrinology and Metabolism, University of Oxford, Oxford, United Kingdom; 9 Department of Clinical Science, Genetic and Molecular Epidemiology, Lund University Diabetes Centre, Lund, Sweden; 10 Novo Nordisk Foundation Center for Protein Research, Faculty of Health and Medical Sciences, University of Copenhagen, Copenhagen, Denmark; 11 Department of Public Health, Faculty of Health and Medical Sciences, University of Copenhagen, Copenhagen, Denmark; 12 Department of Health Technology, Technical University of Denmark, Lyngby, Denmark; 13 Department of Genetic Medicine and Development, University of Geneva Medical School, Geneva, Switzerland; 14 Wellcome Centre for Human Genetics, University of Oxford, Oxford, United Kingdom; 15 Diabetes Endocrinology and Reproductive Biology, Ninewells Hospital and Medical School, University of Dundee, Dundee, United Kingdom; 16 Precision Healthcare University Research Institute, Queen Mary University of London, London, United Kingdom; 17 Metabolomics and Proteomics Core, German Research Center for Environmental Health, Helmholtz Zentrum München, München, Germany; 18 Department of Clinical Medicine, Faculty of Health and Medical Sciences, University of Copenhagen, Copenhagen, Denmark; 19 Medical Department, Zealand University Hospital, Køge, Denmark; 20 Department of General Practice Medicine, Amsterdam UMC, Amsterdam, The Netherlands; 21 Department of Clinical and Biomedical Sciences, University of Exeter College of Medicine & Health, Exeter, United Kingdom; 22 Macleod Diabetes and Endocrine Centre, Royal Devon University Healthcare NHS Foundation Trust, Exeter, United Kingdom; 23 SciLifeLab, Department of Protein Science, KTH - Royal Institute of Technology, Stockholm, Sweden; 24 Internal Medicine, Institute of Clinical Medicine, University of Eastern Finland, Kuopio, Finland; 25 Institute of Neuroscience, National Research Council, Rome, Italy; 26 Academic Department of Clinical Biochemistry, Royal Devon University Healthcare NHS Foundation Trust, Exeter, United Kingdom; 27 Global Development, Sanofi-Aventis Deutschland GmbH, Frankfurt am Main, Germany; 28 Eli Lilly Regional Operations GmbH, Wien, Austria; 29 Center for Clinical Metabolic Research, Herlev and Gentofte University Hospital, Copenhagen, Denmark; 30 Sanofi Partnering, Sanofi-Aventis Deutschland GmbH, Frankfurt am Main, Germany; 31 Department of Epidemiology and Data Science, Amsterdam UMC, Amsterdam, The Netherlands; 32 Department of Cell and Chemical Biology, Leiden UMC, Leiden, The Netherlands; 33 Steno Diabetes Center Copenhagen, Copenhagen, Denmark; 34 Translational and Clinical Research Institute, Faculty of Medical Sciences, Newcastle University, Newcastle upon Tyne, United Kingdom; 35 Biosciences Institute, Faculty of Medical Sciences, Newcastle University, Newcastle upon Tyne, United Kingdom; 36 Population Health and Genomics, Ninewells Hospital and Medical School, University of Dundee, Dundee, United Kingdom; University of Minnesota School of Public Health, UNITED STATES OF AMERICA

## Abstract

Here we report the results from exploratory analysis using a Bayesian network approach of data originally derived from a large North European study of type 2 diabetes (T2D) conducted by the IMI DIRECT consortium. 3029 individuals (795 with T2D and 2234 without) within 7 different study centres provided data comprising genotypes, proteins, metabolites, gene expression measurements and many different clinical variables. The main aim of the current study was to demonstrate the utility of our previously developed method to fit Bayesian networks by performing exploratory analysis of this dataset to identify possible causal relationships between these variables. The data was analysed using the BayesNetty software package, which can handle mixed discrete/continuous data with missing values. The original dataset consisted of over 16,000 variables, which were filtered down to 260 variables for analysis. Even with this reduction, no individual had complete data for all variables, making it impossible to analyse using standard Bayesian network methodology. However, using the recently proposed novel imputation method implemented in BayesNetty we computed a large average Bayesian network from which we could infer possible associations and causal relationships between variables of interest. Our results confirmed many previous findings in connection with T2D, including possible mediating proteins and genes, some of which have not been widely reported. We also confirmed potential causal relationships with liver fat that were identified in an earlier study that used the IMI DIRECT dataset but was limited to a smaller subset of individuals and variables (namely individuals with complete data at pre-defined variables of interest). In addition to providing valuable confirmation, our analyses thus demonstrate a proof-of-principle of the utility of the method implemented within BayesNetty. The full final average Bayesian network generated from our analysis is freely available and can be easily interrogated further to address specific focussed scientific questions of interest.

## Author summary

Bayesian network analysis can be used to identify putative causal relationships between measured variables, including clinical measurements and measurements of genetic and genomic factors. Here we report the results from Bayesian network analysis of data originally derived from a large North European study of type 2 diabetes (T2D). Data were collected for 3029 individuals within 7 different study centres, with the data comprising genotypes, proteins, metabolites, gene expression measurements and many different clinical variables. The original dataset consisted of over 16,000 variables, which were then filtered down to 260 variables for analysis. Even with this reduction, not one individual had complete data for all variables. Using standard methodology it would not be possible to a fit a Bayesian network as it requires complete data. However, using the novel imputation method implemented in the BayesNetty software package, we were able to compute a large average Bayesian network from which we could infer possible associations and causal relationships between variables of interest.

## Introduction

The IMI DIRECT (DIabetes REsearCh on patient straTification) consortium [1, 2] was set up to gather a wide range of data at different study centres throughout Northern Europe in order to study different aspects of type 2 diabetes (T2D). Findings from this dataset have been on topics as diverse as associations with liver fat [3] and inferring regulatory networks [4]. Here, rather than focussing on a specific question related to T2D, we perform exploratory analysis of the dataset to identify possible causal relationships between variables using Bayesian network (BN) methodology as implemented in our own BayesNetty software [5, 6]. BNs can be used to infer possible causal relationships between variables based on their conditional dependencies and independencies, which can be particularly useful in complex biological scenarios with many measured variables. Understanding the relationships between measured biological and clinical variables can inform about underlying biological mechanisms, which may ultimately have clinical implications. The main advantage of our approach is that it can handle mixed continuous/discrete data with missing values, which is vital for these types of multi-omics datasets, as such studies often contain a considerable amount of missing data, with no individuals having complete data for every variable. Our approach also leverages the information provided by genetic variables (which can be considered as causal anchors) to help better resolve the direction of non-genetic edges when fitting the BN, in a conceptually similar (but complementary) approach to Mendelian Randomization [5, 7].

Previous studies focussed on T2D [8, 9] have used colocalization analysis [10] of molecular quantitative trait loci (QTL) to identify a number of potential “effector” variables, including transcripts (gene expression) and protein levels, that may explain the genetic associations seen with T2D. Mahajan *et al*. (2022) [9] identified 97 candidate effector genes, including *ADCY5* and *TCF7L2*, based on circulating plasma proteins (pQTL) and gene expression (eQTL) in diverse tissues, while Vi nuela *et al*. (2020) [8] focussed on expression in human pancreatic islets and showed colocalization between genetic variants influencing T2D or glycemic traits and 47 islet *cis*-eQTL, including *ADCY5*, *DGKB* and *TCF7L2*. Colocalization indicates that the two traits in question most likely share a causal genetic factor but does not identify the directionality of the relationship; it is possible that either trait acts as a mediator for the other, or that the genetic variant has independent horizontal pleiotropic effects on both traits. Therefore, Vi nuela *et al*. (2020) [8] recommend regarding the genes highlighted by coincident GWAS and eQTL signals as candidate effector transcripts that should be further interrogated through experimental approaches that directly test for causality. In their own analysis of the IMI DIRECT dataset, Brown *et al*. (2023) [4] used Bayesian networks applied to triplets of variables (one genetic variant and two molecular phenotypes) to infer the direction of causality, but they did not expand this investigation to include clinical variables such as T2D.

In this current investigation, we fitted a large average BN (see Methods) to the IMI DIRECT dataset after it had been pre-processed to reduce the number of variables down to a manageable size of 260 variables in 3029 individuals. We then used sub-networks (Markov blankets) to focus on variables of interest, such as body mass index (BMI), liver fat and T2D, replicating and confirming many previous findings.

## Results

### Bayesian Network of all variables

**Fig 1:**
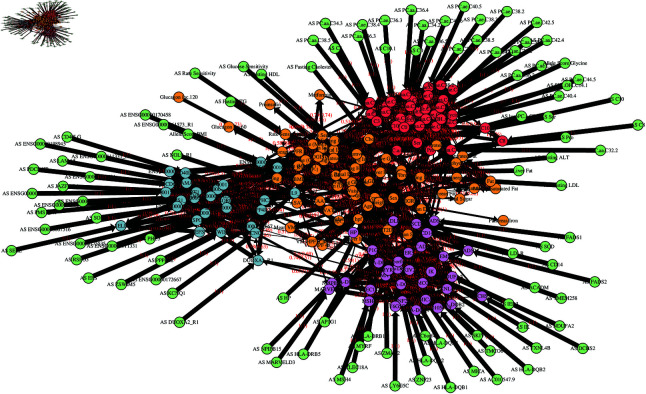
Average BN constructed using imputed data of all variables with strength threshold 0.5. Edges are labelled with the probability that they exist (strength), and, in brackets, the probability that they exist in the shown direction, given that they exist (direction). The thickness of the edges is proportional to the edge strength. The nodes are coloured as follows: red are metabolites; blue (with gene name) are proteins; purple (with gene name) are gene expression measurements; amber are clinical variables; green (prefixed with AS) are allele scores.

We fitted a large average BN (see Methods) to the IMI DIRECT dataset after data pre-processing and plotted it using igraph [11] (Fig 1). The edge strength threshold used for plotting (which represents the posterior probability that a given edge exists) was chosen to be 0.5, the value analytically suggested for this average network by the methods of Scutari *et al*. [12, 13]. Thus, all edges shown in Fig 1 have posterior probabilities greater than 0.5. S1 Table lists all edges from Fig 1 together with their strength and direction values, while S2 Table lists every edge in the average network without using any edge threshold.

The BN in Fig 1 has too many variables (n=260) and edges (n=1123) to make specific inferences via visual inspection, but it nonetheless shows the patterns of overall network structure. We found that variables of the same kind tended to be more connected (e.g. gene expression with gene expression, metabolite with metabolite etc.) (Table 1). Although there were also connections between different kinds of variables, these were less common, so the same kinds of variables tended to visually cluster together when using the automatic plotting feature of igraph. The clinical variables (shown in amber) were often more connected to each other and, to a lesser extent, to other kinds of variables. Allele score variables (shown in green), which represent polygenic risk scores [14] (see Methods), were constrained to be parent variables, as the direction of causal effect only makes biological sense when going from genetic variables to other variables. Allele score nodes in Fig 1 are thus placed around the nodes for their corresponding child variables.

**Table 1:** Percentage of edges in Fig 1 going from/to variables of various types.

	To variable type			
From variable type	clinical	metabolite	protein	expression
clinical	68.30	14.69	7.47	9.54
metabolite	4.96	89.72	2.13	3.19
protein	0.63	1.27	93.67	4.43
expression	2.07	0.52	3.63	93.78

The directionality of the network identified using both strength and direction threshold 0.5 resulted in a mean of 4.53 edges per parent variable pointing towards child variables, while the mean number of connections pointing towards any given child variable was 7.29, suggesting that these child variables were potentially defined by the coordinated causal effects of many other variables. The mean number of connections from clinical, metabolite, protein and expression variables were (6.69, 8.29, 6.58, 6.43) respectively, while the mean number of connections to clinical, metabolite, protein and expression variables respectively were (4.26, 3.18, 5.15, 6.76), suggesting that clinical variables, metabolites and proteins tend to produce more edges than they receive.

From this large network we can focus on variables of interest by plotting their Markov blankets, which consist of all variables that are needed to predict the behaviour of a chosen variable and its children [13].

### Markov blanket for Liver Fat recapitulates previous findings

As a proof-of-principle, we start by investigating a network centred on liver fat, as previously studied by Atabaki *et al*. [3] using the same IMI DIRECT dataset. The focus of the analyses by Atabaki *et al*. [3] was to better understand possible relationships of liver fat with T2D and non-alcoholic fatty liver disease (NAFLD), which frequently co-occur. Atabaki *et al*. [3] also used BN methodology for their analyses focusing on the complete data for their variables of interest including clinical and proteomic measurements (331 individuals with T2D and 964 free from diabetes). In addition, Atabaki *et al*. conducted two-sample bidirectional Mendelian Randomization [15] on some of the edges identified by BN, with genetic instruments leveraged from publicly available sources [3].

Because standard BN implementations usually require observations without missing data, which can be costly and difficult to produce (particularly in the context of clinical data, including data from Electronic Health Records), methods that can cope with missing data like BayesNetty offer a considerable advantage. Incorporation of genetic variables such as allele scores (as is routinely done in BayesNetty) can also help better resolve the direction of edges when fitting the BN. S1 Fig shows the Markov blanket for liver fat derived from Fig 1, which incorporates data from all 3029 individuals available and makes use of genetic variables in the form of allele scores (as outlined in the Methods).

The results of Atabaki *et al*. [3] had suggested that a higher insulin secretion rate (Basal ISR) and excess visceral fat (VAT) accumulation were the most likely clinical factors in the dataset to cause liver fat accumulation and therefore NAFLD. Our selected network for liver fat (S1 Fig, S3 Table) found strong evidence of a causal relationship from VAT to liver fat, with an edge strength value of 0.99 and a direction value of 0.86. There is also strong evidence of a causal relationship of Basal ISR to liver fat with strength and direction values of 0.86 and 0.95 respectively.

Our network identifies both liver iron and VAT to have centre as a parent variable. This node encodes the centre of origin of the samples, thus identifying differences in the measurement of these variables between study centres, but also identifying differences in BMI, as some centres provided samples from individuals with higher values, which introduces a correlation between BMI and centre [1, 2]. We also observed total abdominal adipose tissue (TAAT) and abdominal subcutaneous adipose tissue (ASAT) as causal variables on liver fat (S3 Table), providing an additional causal path from BMI to liver fat. This causal path (Centre → BMI → TAAT → VAT → Liver Fat) is not directly visible in S1 Fig, as BMI does not form part of the Markov blanket for liver fat (which is formally defined as the variable of interest and all parent variables, child variables and variables that are also parents of the child variables [13]), however it can be deduced from the connections from Fig 1 listed in S1 Table.

In summary, BayesNetty was able to provide similar results to those from traditional BN methods, with the added advantage of allowing the inclusion of data (including genetic data) from all participants, even in datasets that include missing data.

### Type 2 Diabetes

**Fig 2:**
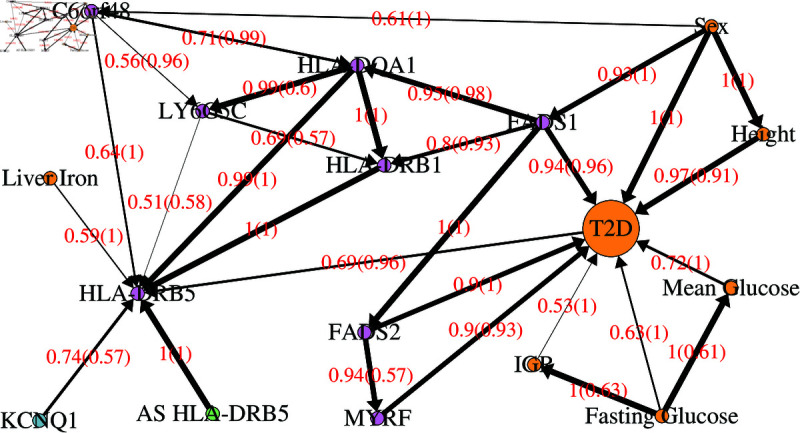
Markov Blanket of type 2 diabetes diagnosis taken from the average BN constructed using imputed data of all variables with strength threshold 0.5. Edges are labelled with the probability that they exist (strength), and, in brackets, the probability that they exist in the shown direction, given that they exist (direction). The thickness of the edges is proportional to the edge strength. The nodes are coloured as follows: red are metabolites; blue (with gene name) are proteins; purple (with gene name) are gene expression measurements; amber are clinical variables; green (prefixed with AS) are allele scores.

Given that the IMI DIRECT cohort was developed to study T2D, including both individuals diagnosed with T2D and those at risk of diabetes, we next evaluated a Markov blanket for T2D (Fig 2). The aim of this investigation was to see to what extent known relationships with T2D could be recapitulated, along with uncovering any novel or unexpected relationships. There were several incoming arrows for the T2D variable (see a full list of all edges in S4 Table). Not unexpectedly, fasting and mean glucose were found as parent (i.e. potential causal) variables for T2D, as T2D is defined by the glucose level in the blood being too high. The variable coding for Impaired Glucose Regulation (IGR) was also found to be a parent variable for T2D. People with IGR have a high blood-glucose level but not high enough to be diagnosed with T2D; this is sometimes referred to as pre-diabetes and is known to be a precursor to a T2D diagnosis [16]. It is known that males have a higher incidence rate of T2D than females and this is shown by the sex variable also being a parent variable of T2D [17]. However, our network suggested this effect was both direct and indirect by also modulating levels of expression for *FADS1*. The level of expression for *FADS2* was also included in the network, identifying the well known involvement of fatty acid levels and the genomic region around those two genes on T2D [18, 19]. Moreover, the network included the expression of *MYRF*. A previous study [20] found a variant in *MYRF* (upstream of *FADS2*) to be associated with lower LysoPC 20:2 and increased risk of T2D. In addition, a multimorbidity study aiming to identify genes acting on multiple diseases was able to identify a cluster of genes including *MYRF* and the *FADS1-FADS2-FADS3* region to be involved in multiple traits such as T2D, coronary artery, BMI and cholesterol among others [21]. Finally, it is perhaps less well known that height has also been associated with T2D, where taller people have been found to have a lower incidence rate of T2D [22–24], perhaps explaining the identification of height as a parent variable of T2D.

Our network for T2D revealed only a single outgoing edge, suggesting a potential causal influence of T2D on *HLA-DRB5* gene expression. While *HLA-DRB5* has been linked to type 1 diabetes (T1D) [25], its involvement in T2D remains unclear, with limited evidence supporting an association between the two [26], presumably in the direction of the gene causing the disease rather than vice versa. We observed a potential causal connection between the abundance of the plasma protein KCNQ1 and the expression of *HLA-DRB5*. The region around the *KCNQ1* gene is well known for the association to T2D [27], pointing to the gene as a likely candidate gene mediating the activity of this locus. However, our network does not report a direct connection between KCNQ1 and T2D, and the connection through *HLA-DRB5* involves an arrow in the “wrong” direction for suggesting a causal effect of gene on disease (going from T2D towards *HLA-DRB5* gene expression rather than vice versa). Overall, these relationships uncovered between *HLA-DRB5*, KCNQ1 and T2D require further investigation and validation, perhaps using different (more targeted) types of data.

It is of interest to investigate to what extent the relationships uncovered by our Bayesian Network approach are supported by evidence from Mendelian Randomization (MR). This is complicated by the fact that many of the relationships we detect are pleoitropic, with the same genetic variant(s) operating through multiple exposures, and with exposures operating through effects on one another. For example, in Fig 2, *FADS1* expression is inferred to operate both directly on T2D and indirectly through *FADS2*, while *FADS2* is inferred to operate both directly on T2D and indirectly through *MYRF*. These types of complicated dependencies between variables causes problems for standard MR approaches[28], which generally require a single route from each exposure to an outcome. This necessitates the use of more sophisticated approaches. We therefore used multivariable MR [29], as well as a recently proposed method, MrDAG [28], to investigate the inferred relationships between *FADS1*, *FADS2*, *MYRF* and T2D, taking advantage of the ability of these methods to operate on summary statistics from large-scale studies, rather than requiring individual-level data. We used genome-wide T2D summary statistics from a large-scale study of 180,834 affected individuals and 1,159,055 controls [9] and downloaded summary statistics for cis-expression quantitative trait loci (cis-eQTL) associations for *FADS1*, *FADS2* and *MYRF* from the eQTLGen consortium [30]. Filtering to only include independent genetic instruments (not in linkage disequilibrium with one another) identified four SNPs that showed significant association with (that thus could be used as instruments for) gene expression, but only one of these (rs198462) also showed significant association with T2D (Table 2. This in itself suggests that MR is unlikely to provide much evidence for causal effects of gene expression on T2D, as the most “basic” implementation of MR essentially boils down to testing for association between the instrument and the outcome[31]. Consistent with this expectation, multivariable MR using the multivariable inverse-variance weighted method implemented within the R package MendelianRandomization provided no significant evidence of causal effects of gene expression on T2D for *FADS1*, *FADS2* and *MYRF* (p-values 0.610, 0.381, 0.232 respectively). Interestingly, univariate analysis using the inverse-variance weighted method did show some evidence for a causal effect of *MYRF* gene expression on T2D (p-value 0.012), although no significant effects were seen at *FADS1* and *FADS2* (p-values 0.292 and 0.550 respectively). This discrepancy between the multivariable and univariate MR analyses can perhaps be attributed to the fact that the variables of interest may indeed be involved in a complicated network of mutual relationships, of which multivariable MR method only provides evidence of effects over and above the effects that are already included in the model—in the case of causal effects between risk factors, estimates represent the direct causal effect of each risk factor on the outcome by a pathway that is not operating via the other risk factors[29].

**Table 2:** Association test p-values from publicly available summary statistics for association between genetic instruments and gene expression or T2D.

SNP	*FADS1*	*FADS2*	*MYRF*	T2D
rs198462	1.74e-23	7.07e-82	3.85e-132	7.70e-03
rs17764389	1.28e-05	1.56e-34	0.338	0.111
rs149778219	1.10e-05	1.45e-03	0.106	0.340
rs2958533	6.41e-03	0.429	6.10e-06	0.888

Analysis using MrDAG also showed little evidence for relationships between *FADS1*, *FADS2*, *MYRF* (considered as exposures) and T2D (considered as the outcome). The only directed edge identified by MrDAG with an estimated probability larger than 0.01 was the edge between *FADS1* and *FADS2*, for which the direction could not be determined (*FADS1* to *FADS2* had edge probability 0.496 while *FADS2* to *FADS1* had edge probability 0.486). The various edges between these particular variables implied by our BN analysis of the IMI DIRECT dataset do not, therefore, seem to be recapitulated in MR analysis of summary statistics from large-scale GWAS. It is possible that there are some unique features of the individual-level IMI DIRECT data that are being captured through our BN approach. Alternatively, it may be that the relationships uncovered in the IMI DIRECT dataset are simply false positives. Ultimately, all of these methods (both the network-based methods and the more traditional MR type methods) are perhaps best considered as exploratory analysis tools, generating putative causal relationships between variables that ideally need further investigation/verification by other means (e.g. experimental laboratory work).

### Body Mass Index

**Fig 3:**
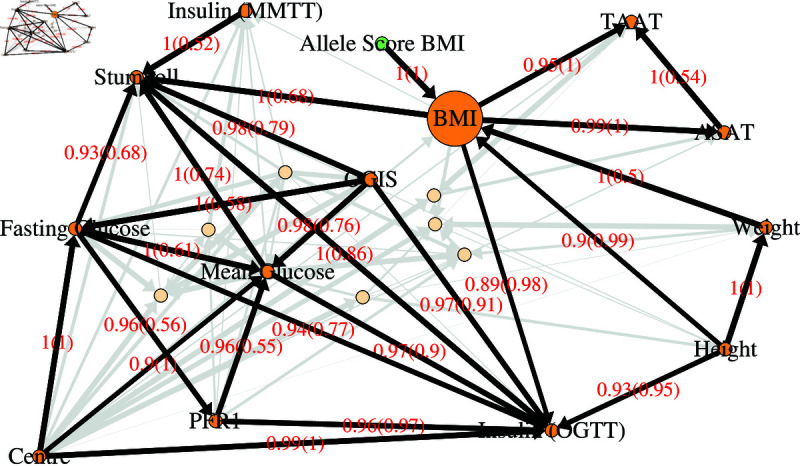
Markov Blanket of BMI. All edges and nodes show a Markov Blanket of BMI taken from the average BN constructed using imputed data of all variables with strength threshold 0.5. Edges and nodes that are not faded show a Markov Blanket of BMI from the average BN with a strength threshold of 0.85 applied instead of 0.5. The thickness of the edges is proportional to the edge strength. Non-faded edges are highlighted in black and labelled in red with the probability that they exist (strength), and, in brackets, the probability that they exist in the shown direction, given that they exist (direction). Nodes are coloured as follows: red are metabolites; blue are proteins; purple are gene expression measurements; amber are clinical variables; green are allele scores.

Next we explore a Markov blanket for body mass index (BMI) (Fig 3, S5 Table). BMI is defined as weight divided by height squared, so strong associations with both weight and height are expected. While the direction value of 0.5 from weight to BMI does not indicate a clear causal relationship, the direction value of 0.99 from height to BMI suggests a potential causal influence. However, this may partly reflect the mathematical structure of BMI rather than a true causal effect. Despite BMI being directly calculated from weight and height, a causal relationship could still exist if these variables co-vary in specific ways across individuals. For instance, previous studies have shown that taller children tend to have higher BMI, while in adults height is inversely associated with BMI—shorter individuals tend to have higher BMI [32]—a pattern that may be captured in the network analysis.

The network suggests that BMI is causal on total abdominal adipose tissue (TAAT) and on abdominal subcutaneous adipose tissue (ASAT), which are essentially measurements of fat around the abdomen. It is generally accepted that obesity causes an increase in these kinds of fat (as discussed, for example, by Verd$\overset{´}{\text{u}}$ *et al*. [33]). A variable for the Stumvoll index is shown; this is designed to measure insulin sensitivity, that is, how sensitive the body is to the effects of insulin, and thus how able to lower blood glucose levels. As BMI is one of the variables used to define the index, a causal relationship from BMI would be expected. However, this connection may also be identifying the known role of BMI on insulin sensitivity. There is also a suggestion of a causal relationship between BMI and insulin from the oral glucose tolerance test (OGTT), which has previously been reported [34]. Overall, the BMI centred network identified the intricate casual relationship between BMI and insulin sensitivity and adipose accumulation, but, with the exception of a genetic variable, the network did not involve any molecular phenotypes.

### Body Mass Index and Type 2 Diabetes

**Fig 4:**
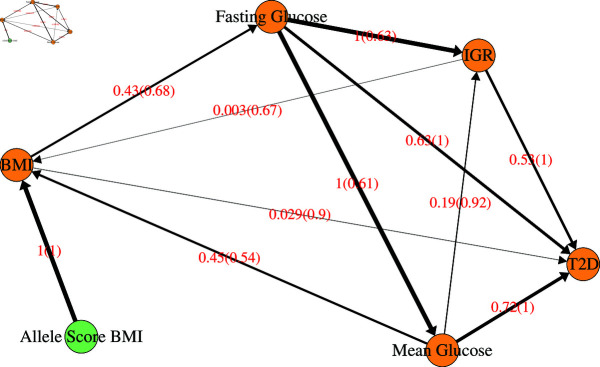
Sub-network taken from the average BN constructed using imputed data of all variables consisting of variables of interest with respect to T2D and BMI. Edges are labelled with the probability that they exist (strength), and, in brackets, the probability that they exist in the shown direction, given that they exist (direction). The thickness of the edges is proportional to the edge strength. The nodes are coloured as follows: amber are clinical variables and green are allele scores.

As there is considerable evidence indicating that obesity is a leading cause of some types of T2D, we attempted to find evidence in our average BN of the possible causal path from BMI to T2D. The exact causal mechanisms linking BMI and T2D are not fully understood and, from this dataset, there was no strong evidence linking them directly or indirectly. There is, however, some weak evidence that there is a causal path via fasting glucose and perhaps mean glucose, both of which appear in the Markov blankets for T2D (Fig 2) and BMI (Fig 3). As well as a direct link between fasting glucose and T2D, Fig 2 also shows an indirect link via IGR. We therefore examined the sub-network comprised of T2D, BMI, fasting glucose, mean glucose, IGR and all edges between them (Fig 4, S6 Table). There have been some studies linking BMI to fasting glucose [35], so, despite the weakness of the edges to/from fasting glucose (0.43) and mean glucose (0.45), these may represent genuine relationships. The direction value of BMI to mean glucose is around 0.5, so there is no strong evidence for the directionality of this edge, while the edge from BMI to fasting glucose has a direction value of 0.68, providing some evidence that the direction of causality is in the direction shown.

The reasons for the apparent weak evidence linking BMI to T2D in this dataset could be numerous. The T2D variable is already adequately described by variables other than BMI, so if there is a causal relationship from BMI to T2D in the dataset, it may well be captured via other variables. As the mechanisms underpinning the involvement of BMI in T2D are complex, it may not be captured in this dataset other than through multiple variables implicating insulin resistance and glucose management, with weaker or no connections between BMI and β-cell function variables. Finally BMI is a constructed variable calculated from weight and height, designed to indicate excess body fat, and it does not take into account other factors implicated in the development of T2D including fat distribution or sex and ethnic differences. For this reason, the use of BMI as a measure of the risk of T2D and other diseases has long been criticised [36]. Ethnic—or other—heterogeneity between participants could potentially be another reason for the lack of strong evidence linking BMI to T2D in this dataset. Thus, BMI may not stand out as a direct biological variable in the causal path with T2D.

### Markov blanket for Centre explains correlations between technical and biological variables

**Fig 5:**
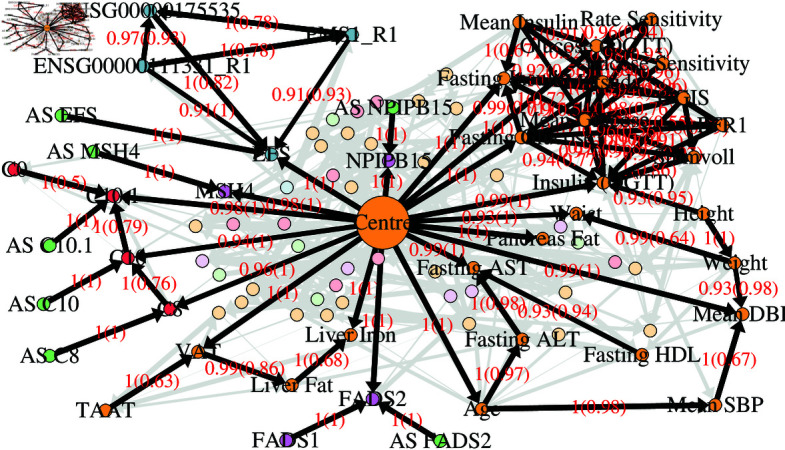
Markov Blanket of centre. All edges and nodes show a Markov Blanket of centre taken from the average BN constructed using imputed data of all variables with strength threshold 0.5. Edges and nodes that are not faded show a Markov Blanket of centre from the average BN with a strength threshold of 0.9 applied instead of 0.5. The thickness of the edges is proportional to the edge strength. Non-faded edges are highlighted in black and labelled in red with the probability that they exist (strength), and, in brackets, the probability that they exist in the shown direction, given that they exist (direction); their connected nodes are also labelled and highlighted. Nodes are coloured as follows: red are metabolites; blue (with gene name) are proteins; purple (with gene name) are gene expression measurements; amber are clinical variables; green (prefixed with AS) are allele scores.

IMI DIRECT is a multi-centre study that used existing population-based cohorts from across Northern Europe. The main study was divided into two cohorts with individuals at risk of diabetes sampled at four centres (Finland, The Netherlands, Denmark and Sweden) and with T2D at six centres (The Netherlands, Denmark, Sweden and UK (Dundee, Exeter, Newcastle upon Tyne)) [1, 2]. As a result, the distribution of some clinically relevant variables is not random. For example, individuals diagnosed with T2D had a mean BMI of $30.5\;{kg/m}^{2}$ while those at risk of diabetes had a mean BMI of $27.9\;{kg/m}^{2}$. We decided to include a variable called “Centre” that identifies the cohort of origin of the data, as it captures subtle biological differences across the dataset. A network focusing on the Centre variable (Fig 5, S7 Table) shows connected clinical and molecular phenotypes associated to these subtle effects. For example, with a very high edge threshold of 0.9, we observed variables relevant for T2D and BMI, included also on Figs 2 and 3. Given that one centre provided samples only for the study of diabetes risk, and three only for the study of T2D, it is not surprising this network partially recapitulates the same connections.

## Discussion

This study aimed to demonstrate the utility of our previously developed method to fit BNs using mixed discrete/continuous data (including missing data) [5, 6] and apply it to an interesting large dataset to investigate possible causal relationships therein. The IMI DIRECT study provided an ideal dataset for this, with many variables that had complete data (e.g. molecular data derived from sequencing methods), but a high missing rate for some sections of the data. The clinical data variables, in particular, had many variables where blocks of individuals had no data, while the non-clinical variables derived from sequencing and high-throughput methods generally had complete data with the exception of a few individuals having no protein data. The missing data pattern for the clinical variables (S2 Fig) shows no individuals with complete data for all variables, however with the use of our imputation method we were able to impute all variables, thus allowing the discovery of possible relationships between variables at an increased power over using a reduced dataset.

The large amount of missing data seen for some clinical variables might be expected to have an impact upon the quality of the imputations for these variables. In our previous work [6] we used computer simulations to explore the performance of our method under varying amounts of missing data (including up to 90% for some variables), and found that our approach was still generally successful in identifying the correct or close-to-correct network structure in terms of connections between variables of interest. As previously noted [6], the goal of our imputation approach is not to impute the missing values as accurately as possible, but rather to impute the missing values in such a way as to allow us *to infer the underlying network* as accurately as possible. For this purpose, the increase in power obtained through performing *any type* of imputation (albeit imprecise), and thus increasing the usable sample size, was found to outweigh any limitations and loss of power due to inaccuracies in the imputed values.

We sought to explore possible causal relationships between variables in the IMI DIRECT diabetes dataset. We did this by firstly fitting a large average BN which revealed that variables of the same kind tend to be more connected to one another, for example, gene expression to gene expression. These results complement those from an earlier study using this same dataset aimed at identifying causal relationships between genetically associated molecular traits (QTLs) [4]. Similar to our current findings, this earlier study found that molecular phenotypes of the same class, e.g. expression-to-expression, show more often dependent relationships between them than phenotypes of different classes. Because the dependencies identified represent causal effects of one phenotype on another, both sets of results likely indicate the fact that variables of the same type are most probably influenced by the same regulatory mechanisms.

We then focussed on the variables of interest by plotting their Markov blanket graphs, in particular focussing on variables for T2D, BMI and liver fat. The graphs confirmed many obvious relationships between variables, such as those between BMI, weight and height. The findings in relation to liver fat corroborate, using a much larger dataset, the main findings of Atabaki *et al*. [3]. The graphs also confirmed many other relationships that have some evidence in the literature, such as *FADS1* and *FADS2* being causal on T2D. However, by design, we did not identify many of the genes reported in other large-scale studies (such as[8]) as being associated with T2D, on account of the fact that, in order to reduce the number of variables to consider to a manageable size, we filtered gene expression variables to choose only those likely to have potentially genetically mediated relationships with the already selected metabolite and protein variables, rather than preferentially selecting gene expression variables that were associated with any particular clinical variable (such as T2D).

A well established cause of T2D is obesity and so, notwithstanding the caveats above, we investigated possible causal routes. There was no strong evidence in the data to support a direct relationship but there was some weak evidence of BMI causing T2D via fasting glucose, possibly implicating the role of insulin resistance. This likely reflects the complexity of the relationship of obesity with T2D and the possibility that there are missing variables that are crucial to describing this relationship.

It was disappointing that not all of the relationships uncovered through BN analysis of the IMI DIRECT dataset could be validated through MR analysis of publicly available summary statistics. A full exploration of all connections identified is beyond the scope of the current investigation and, in any case, would be difficult to implement from a practical point of view, owing to the many pleiotropic and mutually connected relationships that we inferred, along with the requirement to find independent genetic instruments acting on the various exposures. It is possible that there are some unique features of the individual-level IMI DIRECT data that are captured through our BN approach; this, along with the fact that, by design, our approach captures complicated networks of dependencies rather than overall average effects, could also explain why not all of our findings are recapitulated within the literature. BN and MR can be considered as somewhat complementary approaches[5], both of which make various different (largely unverifiable) assumptions and can be sensitive to violations of these assumptions. MR (and variations thereof) tends to be more useful for examining specific hypothesized exposure–outcome relationships when the direction of relationships between variables is already known (or can be plausibly assumed), while BN tends to be more useful for exploring different possible configurations of relationships between all measured variables. Ultimately, all of the approaches considered here are perhaps best considered as exploratory analysis tools, generating putative causal relationships between variables that ideally need further investigation/verification by other means (e.g. experimental laboratory work). However, we note that this may be easier for some types of exposures (e.g. for biological measurements such as gene expression) than for others.

In conclusion, we have applied our BN imputation approach to identify possible causal relationships between variables in a large-scale clinical study of T2D, leading to the replication of many previously identified causal relationships in the final average BN. Our imputation method was vital for the analysis of this large dataset due to the structure of the missing data, and the approach is applicable to large, complex networks containing many hundreds of variables.

## Methods

### BayesNetty software

For details of the methodology implemented in our Bayesian network software package, BayesNetty, please see our earlier publications [5, 6]. The code for BayesNetty is open source, implemented in C++ and is freely available to download from the BayesNetty website and GitHub, where there is also documentation with working examples. The software includes output to plot graphs of the identified BNs using the R package igraph [11], which is used for the plots throughout this manuscript.

### Average networks

An average network, as described by Scutari and Denis [13], is a useful device to account for uncertainty in the direction of edges and in the network structure identified as a whole. To compute the average network, the data is bootstrapped with replacement many times (throughout this manuscript we use 1000 bootstrap replicates) and the best fit network is fitted at each iteration. The best fit network is the directed acyclic graph (DAG) whose network score suggests that it “best” represents the relationships between variables implied by the data; the Bayesian information criterion (BIC) used to construct the network score [13] has the structure of a penalized negative log-likelihood, meaning that models with lower BIC scores are considered to be a better fit, with the score for any hypothesized network inversely related to its posterior probability (calculated under specific distributional assumptions, namely that discrete nodes follow a multinomial distribution and continuous nodes a normal distribution, conditional on their parent nodes).

The number of times that an edge appears between two nodes in each best fit network is recorded, together with its direction. This allows us to calculate the *strength* and *direction* values (between 0 and 1) for each pair of nodes, where the strength is defined as the probability (proportion of times) that an edge appears between the two nodes and the direction is the proportion of times that the edge is in a given direction, given that it exists. We informally refer to these strength and direction values as the “posterior probabilities” of an edge existing, or being in a particular direction (given that it exists), although we note that, strictly speaking, these are not posterior probabilities in the Bayesian sense, because they are not sampled from the posterior distribution of the edges or networks.

If an edge has a high direction value, then it indicates that there may be a causal relationship in that direction, while, if the value is near 0.5, this suggests there is little evidence provided by the data for the direction of the relationship. The resultant average network is given by a table listing all possible edges with their strengths and directions. This typically has many unlikely edges which appeared in only a few bootstrap best fit networks and so have low strength. Thus, to plot the network while including only the most reliable edges, a strength threshold can be used to omit weak edges from the plot. Rather than choosing an ad hoc threshold, a suitable choice of threshold has been proposed based upon statistical arguments [12, 13]. This choice of threshold is used as the default when plotting the average graphs calculated using BayesNetty.

### Imputation of missing data

We previously proposed a novel approach for imputing missing data prior to fitting Bayesian networks, designed to optimise accuracy when determining either the best fit or the average network [6]. Here we use a slightly adapted version of this previous approach. In brief, we use a variant of nearest neighbour imputation [37], where the missing data in one individual is replaced with data from another individual, the *nearest neighbour*. An advantage of this approach is that it can be used with mixed discrete/continuous data, without imposing any direction on the relationships between variables at the imputation stage. We start by fitting an initial best fit network calculated on a dataset where the missing data are replaced with randomly sampled values (with replacement) from the set of non-missing values. The algorithm then proceeds through each individual that has missing data in turn. To decide which variables to use for determining the nearest neighbour for a given “index” individual, we use the initial best fit network. For each variable with missing data in the index individual, a list of all the other variables that have connecting edges and non-missing data for the index individual is constructed. The values of these “nearby” variables are then used to calculate the distance between the index individual and every other individual that has non-missing data both for these nearby variables and for the variable with missing data that needs to be imputed. The individual with the smallest distance is designated as the “nearest neighbour” with respect to that missing variable, and their data for the missing variable is then copied across from the nearest neighbour to the index individual. The process of determining the nearest neighbour and copying across the relevant data is then repeated for each variable that is missing in the index individual. The whole procedure is then repeated for each individual that has missing data.

### IMI DIRECT data

The IMI DIRECT (DIabetes REsearCh on patient straTification) consortium [1, 2] is engaged in research on diabetes and was initially funded by the European Union’s Innovative Medicines Initiative (IMI). The IMI DIRECT data used for the analyses in the current paper consists of data collected at baseline from 7 different centres (Amsterdam, Copenhagen, Dundee, Exeter, Kuopio, Lund and Newcastle upon Tyne) and comprises blood/plasma-based measurements of metabolites, proteins and gene expression, clinical variables and SNP genotypes. There were 795 individuals diagnosed with T2D and 2234 individuals without (albeit at risk of) T2D, giving a total of 3029 individuals with a mean age of 61.7 years (standard deviation 6.9 years), 70.7% of whom were male. Full details of the sample collection, laboratory processing and data availability can be found in the publication by Brown *et al*. (2023) [4].

### Data pre-processing

The initial dataset consisted of 119 metabolites, 377 proteins, 16,219 gene expression variables and 65 clinical variables (which, apart from age and sex, were inverse normal transformed), measured at a single (baseline) timepoint, together with over 81 million imputed SNP genotypes. The metabolites, proteins and gene expression variables were considered primarily as potential mediators for one another (and for the clinical variables). In common with Mendelian Randomization approaches to inferring causality, Bayesian network analysis approaches applied to data from a single timepoint make use of the measured genetic factors as causal anchors to orient the direction of edges in a directed acyclic graph, rather than requiring (or making use of) longitudinal measurements [5]. We restricted our focus to the targeted (rather than untargeted) metabolite data generated by IMI DIRECT in order to limit the number of variables considered. As this was still too large a dataset to perform Bayesian network analyses, due to computational limitations and the potential for a high number of false positive edges [38], it was considered appropriate to first reduce the dataset to a more manageable size, retaining only those variables showing the strongest relationships with genetic factors (which can be used to orient the direction of edges). Firstly, we decided to keep only metabolites and protein variables passing a p-value threshold of 0.01 adjusted for multiple testing using Bonferroni correction for association with at least 20 different SNPs from genome-wide association study (GWAS) analyses. Five genetic principal components (PCs) were included as covariates to model any population stratification/substructure. This GWAS of metabolites and proteins resulted in 34 metabolite and 27 protein variables. Requiring association with at least 20 SNPs (many of which would have been in linkage disequilibrium) helped to decrease the possibility that these SNP associations were spurious artefacts. The 4564 SNPs contributing to these associations were then used to perform association analyses (again including PCs as covariates) for each of the 16,219 gene expression variables, retaining those passing a p-value threshold of 0.01 adjusted for multiple testing using Bonferroni correction. Each gene expression variable with more than one significant SNP was retained for BN analyses, resulting in 33 gene expression variables that would be expected, by design, to have potential genetically mediated relationships with the selected metabolite and protein variables. We note that this procedure necessarily removes many gene expression variables that might be of interest with respect to any particular clinical phenotype (such as T2D, for example). If a particular clinical trait were of interest, then an alternative filtering algorithm, that preferentially retains molecular variables on the basis of their association with the chosen clinical phenotype, might be preferred. However, for the purposes of the current investigation, we were more interested in exploring the relationships between different molecular variables than in singling out a particular clinical variable on which to focus. All 65 clinical variables were kept, including a discrete variable for the administrative centre in which the individual data was gathered.

Weighted allele score variables (polygenic risk scores) were created for each retained metabolite, protein and gene expression variable following standard practice [14]. Specifically, these scores were constructed as the sum of the number of risk alleles possessed at each significant SNP, weighted by their estimated allelic effect (as estimated from the relevant metabolite, protein or gene expression GWAS), while pruning SNPs for linkage disequilibrium (to retain only independent genetic predictors) using standard clumping analysis methods. Use of genetic variables such as allele scores (also known as polygenic risk scores) is an important step to better resolve the direction of edges when fitting a BN. Another allele score was also created for BMI using previously identified significant SNPs [39]. Further allele scores were created for any clinical variables with significant SNPs from GWAS analysis carried out using the IMI DIRECT dataset. The threshold used for significant clinical variable–SNP associations was 0.05 adjusted for multiple testing using a Bonferroni correction. This gave 8 more variables with allele scores (fasting HDL, fasting LDL, fasting TG, fasting ALT, fasting cholesterol, glucose sensitivity, rate sensitivity, and liver fat). No significant genetic associations were identified with diabetes, which is perhaps not surprising given that the many genetic associations with type 2 diabetes previously identified through large-scale GWAS [9] have required sample sizes for discovery in the hundreds of thousands. This resulted in a final dataset of 261 variables, from which one duplicate sex/gender variable was removed, leaving 260.

### Analysis

Before any analyses were performed, the data were preprocessed as described above. The resultant dataset consisted of 260 variables for 3029 individuals, however not a single individual had complete data for every variable. Most of the missing data were for clinical variables (S2 Fig) where some variables had no data for either the cases or controls. All of the data for other variables was complete except for 14 individuals with no protein data. As BNs cannot be fitted to data with missing values, we first used our BayesNetty software to impute the missing data as described previously [6]. The imputed dataset was then used to fit an average BN as described above. In the fitting process some constraints were placed on the edges between variables. Allele score variables were constrained as parents for the variable for which they had been constructed and no other edges were permitted to or from the allele score variables. The variable for sex was constrained to have no parent variables and, as the variable for centre was discrete, it was automatically constrained to have no parent nodes that are continuous. T2D was treated as a continuous trait in order to allow it to have parent nodes that are continuous.

The final average network was used to extract sub-networks known as Markov blankets [12], which consist of one variable of interest and all parent variables, child variables and variables that are also parents of the child variables. The Markov blanket thus consists of all information necessary to infer the variable of interest and its child variables.

### Analysis run times

It took around 9 minutes 45 seconds to impute data for each individual, so for the 3029 individuals this would take over 20 days if ran sequentially, however using a computer cluster it was possible to impute the data in parallel, reducing the computation time to less than one day.

To compute the average network, 1000 best fit networks were computed taking around 12 minutes and 41 seconds each to fit, giving a total time of over 8 days to calculate. Again it was possible to do the computation in parallel reducing the computation time to less than one day.

## Supporting information

S1 Fig Markov Blanket of liver fat. All edges and nodes show a Markov Blanket of liver fat taken from the average BN constructed using imputed data of all variables with strength threshold 0.5. Edges and nodes that are not faded show a Markov Blanket of liver fat from the average BN with a strength threshold of 0.85 applied instead of 0.5. The thickness of the edges is proportional to the edge strength. Non-faded edges are highlighted in black and labelled in red with the probability that they exist (strength), and, in brackets, the probability that they exist in the shown direction, given that they exist (direction); their connected nodes are also labelled and highlighted. Nodes are coloured as follows: red are metabolites; blue are proteins; purple are gene expression measurements; amber are clinical variables; green (prefixed with AS) are allele scores.
(PDF)

S2 Fig Missing data pattern of the clinical variables. Each column represents one of the 3029 individuals and each row represents one of the clinical variables. Black shows non-missing data and light grey shows missing data.
(PDF)

S1 Table Edges from network shown in Fig 1, together with their strength and direction values. (CSV)

S2 Table Edges from full average network, together with their strength and direction values. (CSV)

S3 Table Edges from Liver Fat network shown in S1 Fig, together with their strength and direction values. (CSV)

S4 Table Edges from T2D network shown in Fig 2, together with their strength and direction values. (CSV)

S5 Table Edges from BMI network shown in Fig 3, together with their strength and direction values. (CSV)

S6 Table Edges from network focussed on T2D and BMI shown in Fig 4, together with their strength and direction values. (CSV)

S7 Table Edges from Centre network shown in Fig 5, together with their strength and direction values. (CSV)

S1 Note The DIRECT Consortium. (DOCX)
