# Supplementary figures and images for: Bayesian network imputation methods applied to multi-omics data identify putative causal relationships in a type 2 diabetes dataset containing incomplete data: An IMI DIRECT Study
Source: PLoS Genet. 2025 Jul 15;21(7):e1011776. doi: 10.1371/journal.pgen.1011776 (PMC12279144; doi:10.1371/journal.pgen.1011776)

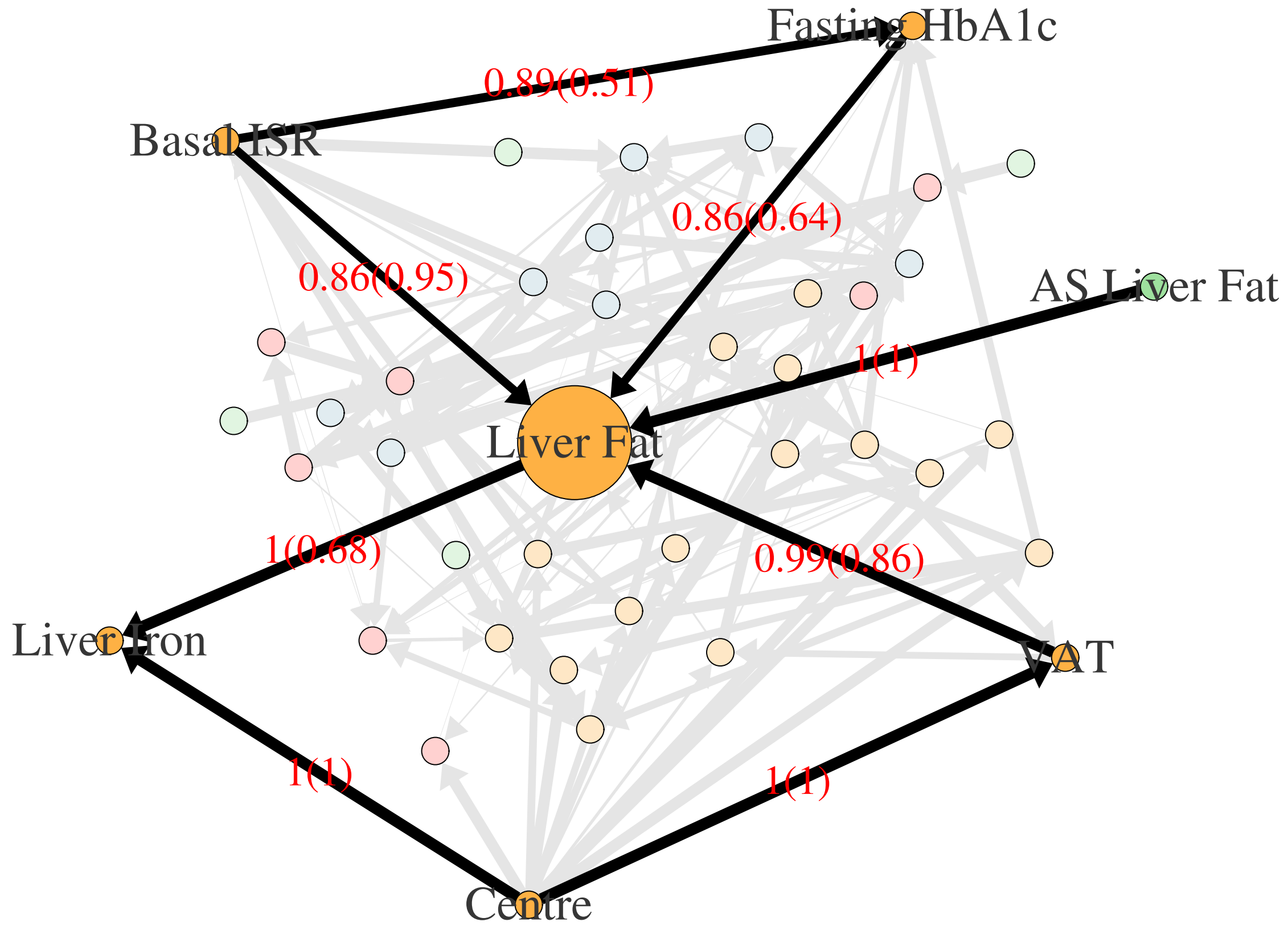

Supplement: S1 Fig — All edges and nodes show a Markov Blanket of liver fat taken from the average BN constructed using imputed data of all variables with strength threshold 0.5. Edges and nodes that are not faded show a Markov Blanket of liver fat from the average BN with a strength threshold of 0.85 applied instead of 0.5. The thickness of the edges is proportional to the edge strength. Non-faded edges are highlighted in black and labelled in red with the probability that they exist (strength), and, in brackets, the probability that they exist in the shown direction, given that they exist (direction); their connected nodes are also labelled and highlighted. Nodes are coloured as follows: red are metabolites; blue are proteins; purple are gene expression measurements; amber are clinical variables; green (prefixed with AS) are allele scores. (PDF) [file pgen.1011776.s001.pdf]

# Missing Clinical Data Pattern

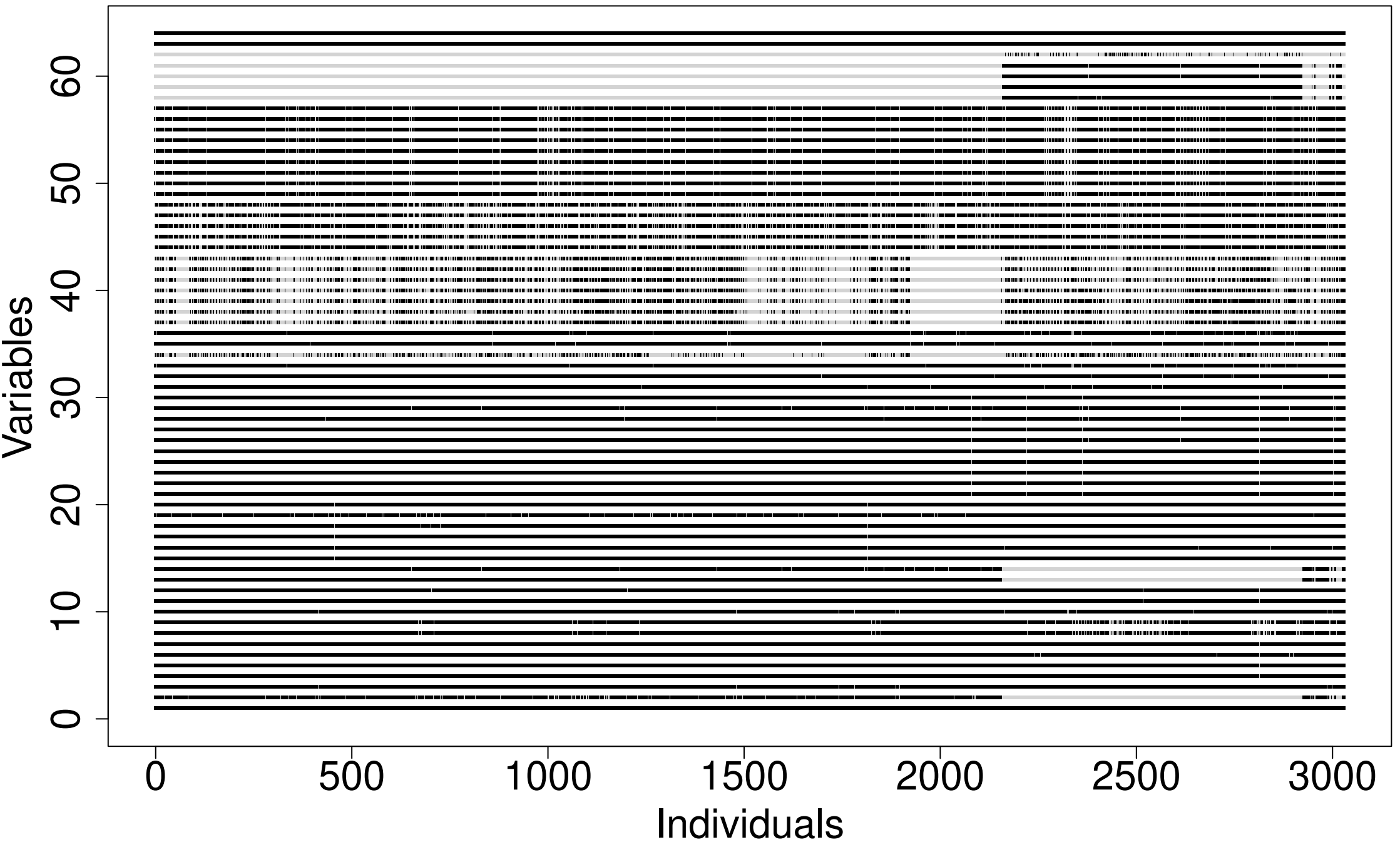

Supplement: S2 Fig — Each column represents one of the 3029 individuals and each row represents one of the clinical variables. Black shows non-missing data and light grey shows missing data. (PDF) [file pgen.1011776.s002.pdf]
