# Supplementary material for: Bayesian network imputation methods applied to multi-omics data identify putative causal relationships in a type 2 diabetes dataset containing incomplete data: An IMI DIRECT Study
Source: PLoS Genet. 2025 Jul 15;21(7):e1011776. doi: 10.1371/journal.pgen.1011776 (PMC12279144; doi:10.1371/journal.pgen.1011776)
Supplement: S1 Note — (DOCX) [file pgen.1011776.s010.docx]

**The DIRECT Consortium**

Jonathan Adam^1^, Kristine H. Allin^2^, Anna A. Artati^3^, Natalie Atabaki^4^, Karina Banasik^5,6^, Anna Barnett^7^, Jimmy D. Bell^8^, Joline W. Beulens^9^, Susanna Bianzano^10^, Roberto Bizzotto^11^, Amelie Bonnefond^12^, Caroline Anna A. Brorsson^5,6^, Andrew A. Brown^13^, Søren Brunak^5,6^, Marc Clos-Garcia^2^, Matilda Dale^14^, David Davtian^13^, Adem Y. Dawed^13^, Christiane Dings^15^, Louise Donnelly^13^, Line Engelbrechtsen^16^, Rebeca Eriksen^17^, Yong Fan^18^, Juan Fernandez^19^, Ian M. Forgie^13^, Paul W. Franks^4^, Philippe Froguel^12,20^, Gary Frost^17^, Johann Gassenhuber^21^, Giuseppe N. Giordano^4^, Toni Giorgino^22^, Lenka Groeneveld^9^, Valborg Gudmundsdóttir^23^, Ramneek Gupta^24^, Mark Haid^25^, Torben Hansen^2^, Tue H. Hansen^2^, Andrew T. Hattersley^26^, Ragna Haussler^27^, Anita M. Hennige^28^, Anita V. Hill^29^, Reinhard W. Holl^30^, Mun-Gwan Hong^27^, Michelle Hudson^29^, Bernd Jablonka^31^, Ulrik Plesner Jacobsen^32^, Christopher Jennison^33^, Angus G. Jones^26^, Tugce Karaderi^5,6^, Jane Kaye^34^, Maria Klintenberg^35^, Robert W. Koivula^36,37^, Tarja Kokkola^38^, Teemu Kuulasmaa^39^, Markku Laakso^38^, Thorsten Lehr^15^, Agnete Troen T. Lundgaard^5,6^, Liwei Lyu^2^, Anubha Mahajan^19^, Andrea Mari^11^, Gianluca Mazzoni^5,6^, Mark I. McCarthy^36,19^, Timothy J. McDonald^26,40^, Nicky McRobert^36^, Pascal Mutie^4^, Rachel Nice^40^, Colin N. Palmer^13^, Francois Pattou^41^, Imre Pavo^42^, Ewan R. Pearson^13^, Oluf Pedersen^2^, Helle K. Pedersen^5,6^, Mandy H. Perry^40^, Cornelia P. Prehn^25^, Anna Ramisch^43^, Simon Rasmussen^5,6^, Violeta Raverdi^41^, Martin Ridderstråle^44^, Hartmut Ruetten^45^, Femke Rutters^9^, Jochen M. Schwenk^27^, Sapna Sharma^46^, Iryna Sihinevich^15^, Roderick Slieker^47,48^, Leen M. 't Hart^49,50^, Cecilia Engel E. Thomas^5,6,27^, Melissa K. Thomas^51^, Elizabeth Louise L. Thomas^8^, Barbara Thorand^52^, Andrea Tura^11^, Sabine van Oort^53^, Jagadish Vangipurapu^38^, Henrik Vestergaard^54^, Ana Viñuela^55^, Josef K. Vogt^56^, Mark Walker^57^, Agata Wesolowska-Andersen^36^

^1^Research Unit of Molecular Epidemiology, Institute of Epidemiology, Helmholtz Zentrum München, Neuherberg, Germany, ^2^Novo Nordisk Foundation Center for Basic Metabolic Research, Faculty of Health and Medical Sciences, University of Copenhagen, Denmark, ^3^Metabolomics and Proteomics Core (MPC) Helmholtz Zentrum München, German Research Center for Environmental Health, Ingolstädter Landstraße 1, 85764 Neuherberg, Germany, ^4^Genetic and Molecular Epidemiology Unit, Lund University Diabetes Centre, Department of Clinical Sciences, CRC, Lund University, SUS, Malmö, Sweden, ^5^Section for Bioinformatics, Department of Health Technology, Technical University of Denmark, Kgs. Lyngby, Denmark, ^6^Disease Systems Biology Program, Novo Nordisk Foundation Center for Protein Research, Faculty of Health and Medical Sciences, University of Copenhagen, Copenhagen, Denmark, ^7^NHS Research Scotland Diabetes, School of Medicine, University of Dundee, United Kingdom, DD1 9SY, ^8^Research Centre for Optimal Health, Department of Life Sciences, University of Westminster, London, United Kingdom, ^9^Department of Epidemiology and Data Science, Amsterdam UMC, Amsterdam, The Netherlands, ^10^Boehringer Ingelheim International GmbH, Therapeutic Area CardioMetabolism and Respiratory Medicine, Ingelheim am Rhein, Germany, ^11^CNR Institute of Neuroscience, Padova, Italy, ^12^INSERM UMR 1283, CNRS UMR 8199, European Genomic Institute for Diabetes (EGID), Institut Pasteur de Lille, University of Lille, Lille University Hospital, Lille, F-59000, France., ^13^Population Health & Genomics, School of Medicine, University of Dundee, Dundee, United Kingdom, ^14^Internal Medicine, Institute of Clinical Medicine, University of Eastern Finland, Kuopio, Finland, ^15^Clinical Pharmacy, Saarland University, Saarbrücken, Germany, ^16^Novo Nordisk Center for Basic Metabolic Research, Section of Metabolic Genetics, Faculty of Health and Medical Science, University of Copenhagen, Copenhagen, Denmark, ^17^Section for Nutrition Research, Faculty of Medicine, Imperial College London, London, United Kingdom, ^18^Novo Nordisk Foundation Center for Basic Metabolic Research, Faculty of Health and Medical Science, University of Copenhagen, Copenhagen, Denmark, ^19^Wellcome Centre for Human Genetics, University of Oxford, Oxford, United Kingdom, ^20^Department of Metabolism, Digestion and Reproduction, Imperial College London, London, United Kingdom., ^21^TMED, Sanofi-Aventis Deutschland GmbH, Frankfurt, Germany, ^22^Biophysics Institute (IBF-CNR), National Research Council of Italy, Milan, Italy, and Department of Biosciences, University of Milan, Milan, Italy, ^23^Section for Bioinformatics, Department of Health Technology, Technical University of Denmark, Kgs. Lyngby, Denmark; Disease Systems Biology Program, Novo Nordisk Foundation Center for Protein Research, Faculty of Health and Medical Sciences, University of Copenhagen, Copenhagen, Denmark, ^24^Novo Nordisk Research Centre Oxford, Oxford, United Kingdom, ^25^Research Unit Molecular Endocrinology and Metabolism, Genome Analysis Center, Helmholtz Zentrum München, German Research Center for Environmental Health, Ingolstädter Landstraße 1, 85764 Neuherberg, Germany, ^26^Institute of Clinical and Biological Sciences, University of Exeter Medical School, Exeter, United Kingdom, ^27^Affinity Proteomics, Science for Life Laboratory, School of Engineering Sciences in Chemistry, Biotechnology and Health, KTH - Royal Institute of Technology, Solna, Sweden, ^28^Boehringer Ingelheim International GmbH, Medicine Cardiometabolism and Respiratory, Biberach an der Riss, Germany, ^29^NIHR Exeter Clinical Research Facility, University of Exeter Medical School, Exeter, United Kingdom, ^30^Institute for Epidemiology and Medical Biometry, ZIBMT, University of Ulm, Ulm, Germany, ^31^Strategy & Innovation, Sanofi-Aventis Deutschland GmbH, Frankfurt, Germany, ^32^University of Copenhagen, Novo Nordisk Foundation Center for Protein Research, Blegdamsvej 3B, building 6, 06-2-17, DK-2200 Copenhagen N, ^33^Department of Mathematical Sciences, University of Bath, Bath, United Kingdom, ^34^Centre for Health, Law and Emerging Technologies (HeLEX), Faculty of Law, University of Oxford, Oxford, United Kingdom, and Centre for Health, Law and Emerging Technologies (HeLEX), Melbourne Law School, University of Melbourne, Carlton, Victoria, Australia, ^35^VO Endokrinologi, Enheten för diabetesstudier Lasarettsgatan 15, Skånes Universitetssjukhus i Lund, Sweden, ^36^Oxford Centre for Diabetes, Endocrinology and Metabolism, Radcliffe Department of Medicine, University of Oxford, Oxford, United Kingdom, ^37^Department of Clinical Sciences, Lund University Diabetes Centre, Genetic and Molecular Epidemiology Unit, CRC, Skåne University Hospital Malmö, Building 91, Level 10, Jan Waldenströms gata 35, SE-205 02 Malmö, Sweden, ^38^Institute of Clinical Medicine, Internal Medicine, University of Eastern Finland, Kuopio, Finland, ^39^Institute of Biomedicine, Bioinformatics Center, University of Eastern Finland, 70210 Kuopio, Finland, ^40^Blood Sciences, Royal Devon and Exeter NHS Foundation Trust, Exeter, United Kingdom, ^41^Inserm, Univ Lille, CHU Lille, Lille Pasteur Institute, EGID, Lille, France, ^42^Eli Lilly Regional Operations GmbH, Vienna, Austria, ^43^Department of Genetic Medicine and Development, University of Geneva Medical School, Geneva, Switzerland, ^44^Clinical Obesity, Lund University Diabetes Centre, Department of Clinical Sciences, Clinical Research Centre, Lund University, Skåne University Hospital Malmö, Malmö, Sweden, ^45^Boehringer Ingelheim International GmbH, ^46^Research Unit of Molecular Epidemiology, Institute of Epidemiology, German Research Center for Environmental Health, Helmholtz Zentrum München, Germany, ^47^Department of Epidemiology and Biostatistics, Amsterdam UMC, Amsterdam, The Netherlands, ^48^Department of Cell and Chemical Biology, Leiden University Medical Center, Leiden, The Netherlands, ^49^Department of Cell and Chemical Biology, Leiden University Medical Center, Leiden, The Netherlands., ^50^Department of Epidemiology and Data Science, Amsterdam UMC, Amsterdam, The Netherlands., ^51^Eli Lilly and Company, Indianapolis, Indiana, USA, ^52^Institute of Epidemiology, Research Unit of Diabetes Epidemiology, Helmholtz Zentrum München, German Research Center for Environmental Health, Neuherberg, Germany, and German Center for Diabetes Research (DZD), München-Neuherberg, Germany, ^53^Department of Epidemiology & Biostatistics, Amsterdam UMC, Amsterdam, The Netherlands, ^54^Bornholms Hospital, Rønne, Denmark; Novo Nordisk Foundation Center for Basic Metabolic Research, Faculty of Health and Medical Sciences, University of Copenhagen, Denmark, ^55^Population Health and Genomics, Ninewells Hospital & Medical School, University of Dundee, United Kingdom, ^56^Novo Nordisk Center for Basic Metabolic Research, Faculty of Health and Medical Science, University of Copenhagen, Copenhagen, Denmark, ^57^Translational and Clinical Research Institute, Faculty of Medical Sciences, Newcastle University, Newcastle, United Kingdom
